# Mammalian *BEX*, *WEX *and *GASP *genes: Coding and non-coding chimaerism sustained by gene conversion events

**DOI:** 10.1186/1471-2148-5-54

**Published:** 2005-10-12

**Authors:** Eitan E Winter, Chris P Ponting

**Affiliations:** 1 MRC Functional Genetics Unit, University of Oxford, Department of Human Anatomy and Genetics, South Parks Road, Oxford OX1 3QX, UK

## Abstract

**Background:**

The identification of sequence innovations in the genomes of mammals facilitates understanding of human gene function, as well as sheds light on the molecular mechanisms which underlie these changes. Although gene duplication plays a major role in genome evolution, studies regarding concerted evolution events among gene family members have been limited in scope and restricted to protein-coding regions, where high sequence similarity is easily detectable.

**Results:**

We describe a mammalian-specific expansion of more than 20 rapidly-evolving genes on human chromosome Xq22.1. Many of these are highly divergent in their protein-coding regions yet contain a conserved sequence motif in their 5' UTRs which appears to have been maintained by multiple events of concerted evolution. These events have led to the generation of chimaeric genes, each with a 5' UTR and a protein-coding region that possess independent evolutionary histories. We suggest that concerted evolution has occurred via gene conversion independently in different mammalian lineages, and these events have resulted in elevated G+C levels in the encompassing genomic regions. These concerted evolution events occurred within and between genes from three separate protein families ('brain-expressed X-linked' [*BEX*], WWbp5-like X-linked [*WEX*] and G-protein-coupled receptor-associated sorting protein [*GASP*]), which often are expressed in mammalian brains and associated with receptor mediated signalling and apoptosis.

**Conclusion:**

Despite high protein-coding divergence among mammalian-specific genes, we identified a DNA motif common to these genes' 5' UTR exons. The motif has undergone concerted evolution events independently of its neighbouring protein-coding regions, leading to formation of evolutionary chimaeric genes. These findings have implications for the identification of non protein-coding regulatory elements and their lineage-specific evolution in mammals.

## Background

Discriminating mutations arising during the evolution of mammals which were selectively neutral from those which were adaptive is an important challenge for the current genomic era. In the main, beneficial mutations in mammalian genomes appear to have been gene duplication, rapid sequence divergence, and alteration in gene expression levels [1-4].

An additional lineage-specific mutational process which is also a substrate for selection is concerted evolution, via either unequal crossing-over or gene conversion [5,6]. Non-allelic gene conversion occurs during non-reciprocal homologous recombination when sequence-similar paralogues are misaligned. Converting sequences are often short, in the order of hundreds of basepairs, and when frequent and sustained can lead to the homogenisation of multigene family sequences [7], as observed for mammalian histone [8] and Hsp70 [9] genes. During gene conversion, repair at mismatched positions appears to be biased towards retention of G or C bases which leads to elevation in G+C nucleotide content [10].

Non-allelic gene conversion thus results in phylogenetic trees which display significantly greater proximity between a species' gene paralogues than for gene orthologues of a sister species [11]. Such phylogenetic relationships, however, are also indicative of lineage-specific gene duplication events. Nevertheless, when the affected genes are widely-spread on the genome these relationships are usually indicative of non-allelic gene conversion. This is because gene duplication most frequently results in tandem consecutive genes along the chromosome.

Gene conversion events are expected to occur mostly between protein-coding sequences of genes. This expectation arises from sequence conservation being, in general, highest in protein-coding regions, intermediate in untranslated regions (UTRs), and lowest within introns and intergenic regions [12]. Gene conversion thus is not expected between sequence-dissimilar and non-homologous genes.

Here, we describe genes whose evolution defies these expectations. We present evidence for gene conversion events between mammalian-specific genes which encode sequence-dissimilar and possibly non-homologous, proteins. These conversion events occurred not within their protein-coding or 3' UTR sequences, but rather within their 5' UTRs and upstream regions. We suggest that the occurrence of concerted evolution events during mammalian evolution led to multiple chimaeric genes, with 5' UTR and protein-coding sequences possessing different evolutionary pedigrees.

These proposed events occurred within and between genes from three separate families ('brain-expressed X-linked' [*BEX*], WWbp5-like X-linked [*WEX*] and G-protein-coupled receptor-associated sorting protein [*GASP*]), all of which contain single protein-coding exons. *Bex1*, *-2 *and *-3 *are 'brain-expressed X-linked genes' whose intracellular products bind protein [13]. BEX1 and BEX2 bind the olfactory marker protein (OMP) [14,15] whereas BEX3 binds the p75 neurotrophin receptor (p75NTR) [16], and the second mitochondria-derived activator of caspase (Smac) [17,18], as well as self-associating [16]. WEX proteins include WWbp5 which is a poorly-understood WW domain binding protein [19]. GASP-1 and -2 are G-protein-coupled receptor (GPCR) associated sorting proteins (GASPs) which bind to the COOH-termini of various GPCRs and modulate their endocytic sorting to lysosomes [20,21].

Genes from these three families are all tightly-clustered within a mammal-specific ~2 Mb region of human chromosome Xq22.1-q22.2. We find that these genes all arose during early eutherian evolution and have experienced substantial sequence divergence thereafter. Their localisation to brain tissues, and their unusual and rapid evolution, are thus consistent with their involvement in the evolution of innovative brain cortical structures among eutherian mammals.

## Results and discussion

### A 2.3 Mb region of human Xq22.1-q22.2 is specific to placental mammals

**Figure 1:**
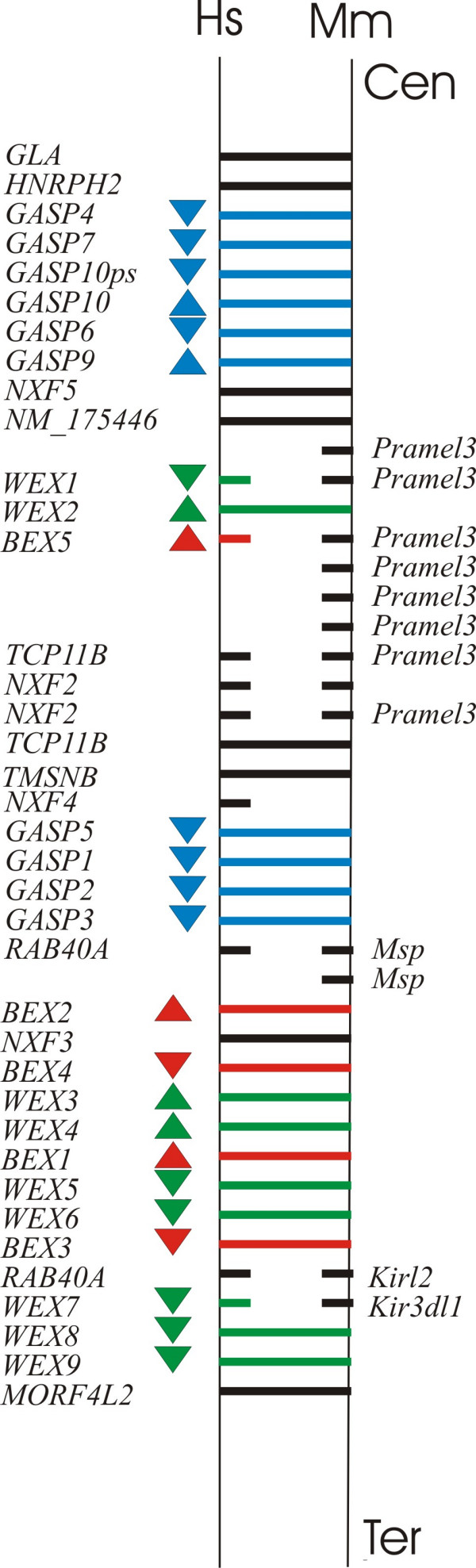
**Protein-coding gene order in human chromosome Xq22.1-q22.2 (~2.3 Mb) and in its mouse orthologous region. **Transcriptional orientations are indicated by filled arrow heads. Human- or mouse- specific genes are indicated by short bars. Abbreviations: Cen, Centromere; Ter, Terminal. Gene names are abbreviated as in Table 1.

Our studies focused on a 2.3 Mb region of human and mouse X chromosomes that encode a collinear arrangement of multiple genes from *BEX*, *WEX *and *GASP *families (Figure 1; Table 1). This entire region has a counterpart in neither the chicken nor the metatherian (marsupial) *Monodelphis domestica*. Orthologues of two genes that flank the region, *Gla *(encoding α-galactosidase; NM_013463) and *Glra4 *(encoding glycine receptor subunit α4; NM_010297), are separated by less than 4 Kb on chicken chromosome 4, and 10 Kb on *Monodelphis *scaffold 13561 (which is expected to lie within the long arm of its X chromosome [22]). These two non-eutherians' intergenic regions are both devoid of sequences homologous to *BEX*, *WEX *and *GASP *genes, and of assembly gaps greater than 100 b.

**Table 1:** Gene and transcript annotation of protein-coding genes located on human chromosome Xq22.1-q22.2. Human *WEX1 *has arisen from duplication of *WEX2*, and human *BEX5 *is absent from the mouse genome. ^a ^Levels of transcript expression in the brain are based on Microarray gene hybridization results [57]. Accession codes and expression tags are shown in parentheses. A gene expression in the brain is absent (-), present (+), or high relative to other tissues (++). Abbreviations: *GLA*, Galactosidase, alpha; *HNRPH2*, Heterogeneous nuclear ribonucleoprotein H2; *GASP*, G protein-coupled receptor-associated sorting; *NXF*, Nuclear RNA export factor; *PRAMEL3*, Preferentially expressed in melanoma like 3; *WEX*, WWbp5-like gene family; *BEX*, Brain-expressed X-linked; *TCP11B*, T-complex 11 B; *TMSNB*, Thymosin, beta, identified in neuroblastoma; *RAB40A*, Ras oncogene family member; *MSP*, Microsomal signal peptidase 23 kDa subunit (SPase 22 kDa subunit); *KIRL2*, Killer immunoglobulin-like receptor-like 2; *KIR3DL1*, Killer immunoglobulin-like receptor 3DL1; *MORF4L2*, Mortality factor 4 like 2;

RefSeq Gene	RefSeq Protein	Synonyms	Gene name	Gene Start	Gene End	Transcriptional orientation	Expression in the brain^a ^(accession/expression-tag)	BGW element
NM_000169	NP_000160		GLA	99424654	99434808	-	+ (GLA/214430_at)	
NM_019597	NP_062543		HNRPH2	99435140	99440975	+	+ (HNRPH2)	+
NM_152583 (BC036206)	NP_689796	Q8K2R3	GASP4	99512319	99560303	+	+ (gnf1h06491_at)	+
NM_016608	NP_057692	Alex1/Q9P291	GASP7	99577371	99581530	+	+ (ARMCX1)	+
			GASP10ψ	99624346	99624903	+	N/A	+
NM_019007	NP_061880	Q9NWJ3	GASP10	99641972	99644848	-	+ (FLJ20811)	+
NM_016607	NP_057691	Alex3, Q9UH62	GASP6	99649976	99654688	+	+ (ARMCX3)	+
NM_177949	NP_808818	Alex2, O60267	GASP9	99682129	99686692	-	+ (ARMCX2)	+
NM_032946	NP_116564		NXF5	99858942	99884406	-	N/A	
AK094141	XP_040486		KIAA1789	99910469	99958857	-	N/A	
NM_080390 (AL540086)	NP_525129	my048	WEX1	100152572	100154538	+	++ (AF063606)	+
BC071675	N/A		WEX2	100167309	100169228	-	N/A	+
BC042818	N/A		BEX5	100180542	100182792	-	N/A	+
NM_017809 (BX647232)	NP060279		NXF2	100242137	100353489	+	+ (NXF2/220257_x_at)	
NM_017809 (BX647232)	NP060279		NXF2	100387175	100498589	-	+ (NXF2/220257_x_at)	
NM_021992	NP_068832		TMSNB	100540461	100543504	-	++ Fetal brain (205347_s_at)	
NM_207512	NP_997395		NXF4	100576750	100598478	+	N/A	
NM_022838	NP_073749	Q9H969	GASP5	100626014	100630940	+	+ (FLJ12969/219335_at)	
NM_014710	NP_055525	Q43168	GASP1	100678299	100685235	+	++ (GASP/204793_at)	
NM_138437 (AK123832)	NP_612446	Q96D09	GASP2	100739148	100744517	+	N/A	
NM_030639	NP_085142	Q9BE11	GASP3	100747511	100779225	+	+ (KIAA1701/213709_at)	
			Rab40A-like	100964038	100965103	+	++ (108_g_at)	
NM_018476 (BX098763)	NP_060946	BEX1, HBEX2	BEX2	101089445	101090956	-	++ (BEX1/218332_at)	+
NM_022052	NP_071335		NXF3	101102606	101119879	-	- (NXF3/220110_s_at)	
AK000959	N/A	Bex4	BEX4	101241922	101243977	+	++ (BEXL1/ 215440_s_at, 40916_at**)	
NM_153333 (AL709034)	NP_699164	Wex3	WEX3	101279783	101281945	-	N/A	+
AL550633	N/A	Wex4	WEX4	101300507	101303513	-	++ (gnf1h07374_s_at, gnf1h07373_at)	+
NM_032621 (AL520932)	NP_116010	Bex2, mouseBex1	BEX1	101336132	101337663	-	N/A	
NM_152278 (H09952)	NP_689491	Wex5	WEX5	101357022	101359108	+	++ (gnf1h06733_at)	+
NM_016303 (BF028506)	NP_057387	Wex6	WEX6	101383280	101385238	+	+ (WBP5 / 217975_at)	+
NM_206915	NP_996798	Ngfrap1	BEX3	101403125	101404856	+	++ (NGFRAP1/217963_s_at)	+
NM_080879	NP_543155		RAB40A	101526538	101546274	-	N/A (RAB40A, 217589_at, 33477_at)	
NM_024863 (AK024827)	NP_079139	Wex7	WEX7	101603016	101614300	+	++ (FLJ21174)	+
NM_032926	NP_116315	Wex8	WEX8	101634691	101636711	+	++ (MGC15737)	+
NM_004780	NP_004771	TCEAL1	WEX9	101655759	101657725	+	++ (TCEAL1)	+
NM_012286	NP_036418		MORF4L2	101702290	101713453	-	+ MORF4L2	

Human Xq22.1-q22.2 thus appears to be an innovation of eutherian (placental) mammals. This is surprising since chromosome Xq initially arose after the divergence of the lineages leading to modern birds and mammals, but prior to the metatherian-eutherian split [23]. The observation however, provides an excellent opportunity to investigate issues underlying sequence innovation and functional innovation. We were interested in three interrelated questions: (i) Which evolutionary processes led to the origin of these genes? (ii) Are these genes' functions similar, and are they related to physiological or anatomical innovations in placental mammals? and, (iii) Why are these newly-acquired genes restricted to this one chromosomal region, rather than being dispersed throughout the remainder of the X chromosome or elsewhere?

### BEX and WEX proteins are diverged homologues

We investigated the evolutionary origins of *BEX*, *WEX *and *GASP *genes using database searches of known protein and nucleotide sequences. We concluded that mammalian X-linked *GASP *genes are members of an ancient family that are discernible in early-branching bony vertebrates such as teleost fish (FLJ20811; NM_212853). By contrast, from the results of BLAST and PSI-BLAST [24] searches, *BEX *or *WEX *gene homologues appear to be absent outside of eutherian mammals. The order of *BEX*, *WEX *and *GASP *genes is conserved between human, rodent and canine X chromosomes and thus must have been present in their common ancestor.

Nevertheless, despite their sequence divergence we find that BEX and WEX protein sequences are homologous. Using COMPASS [25], significant similarity (Smith-Waterman score 82; *E *= 4.4 × 10^-8^) between a multiple sequence alignment of 25 sequences from the BEX protein family, and an alignment of 18 WEX sequences was observed. We thus predict that WEX and BEX proteins arose from a single ancestral gene early in eutherian evolutionary history, but diverged as separate families thereafter by numerous events of gene duplication. This is also consistent with *BEX *and *WEX *gene families possessing a common gene structure, namely three exons, with the most 3' of these always containing the entire protein-coding sequence.

### 5' UTR sequences of *BEX*, *WEX *and *GASP *genes are homologous

Despite the lack of evidence for homology between BEX or WEX, and GASP proteins, we were surprised to observe highly-similar sequences within the 5' UTRs of *BEX *and *GASP *genes. For example, a 383 b sequence (human chrX:101090862–101091244) which overlaps and extends the first non-coding exon of *BEX2 *is 91% identical to a region straddling the first exon of human *GASP4*.

**Figure 2:**
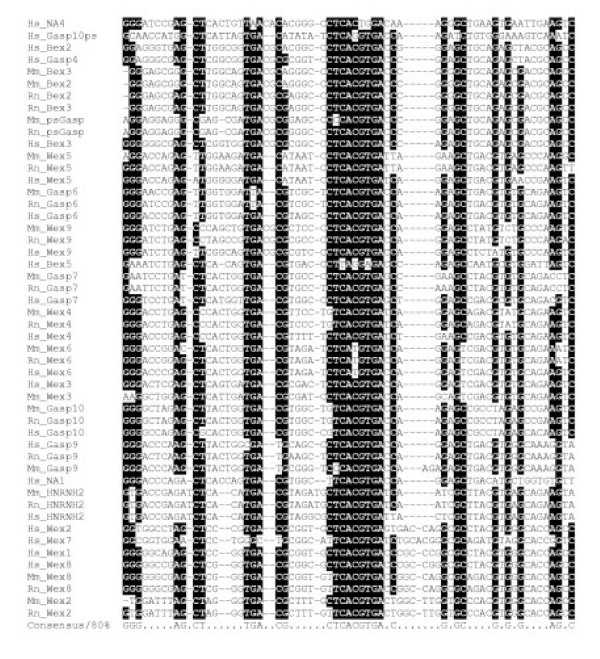
**Multiple sequence alignment of a conserved region of BGW sequence elements. **Shaded if columns are conserved in at least 80% of sequences. Abbreviations: Hs, *Homo sapiens*; Mm, *Mus musculus*; Rn, *Rattus norvegicus*; NA, sequences not 5' to an identifiable gene structure.

Using MEME [26] we searched the first 5' UTR exons of human, mouse and rat *BEX*, *WEX *and *GASP *gene sequences for conserved DNA motifs. A highly significant DNA motif (*E*-value = 5.8 × 10^-390^, width = 57 bases) was identified by MEME in 37 of these 50 sequences. Using BLASTn searches of genomic sequences, we extended the width and breadth of these motifs, which we call BGW (Bex/GASP/Wex) elements (Figure 2). The program MAST [27] and the position-specific matrix of the MEME BGW motif was then used to search the NCBI non-redundant DNA database for significantly scoring DNA alignments. This search yielded statistically significant hits (*E *< 10^-7^) for 18 genomic sequences on human, mouse or rat chromosomes X, all of which represent the 5' UTRs of *BEX*, *WEX *and *GASP *genes. No significant alignments were observed to non-mammalian sequence or to mammalian sequence present outside of the X chromosome.

These BGW motifs are not randomly positioned with respect to coding sequences: 18 of the 23 human homologues occur within the 5' UTRs of *BEX *or *WEX *or *GASP *family genes (Table 1). Of the remaining 5, 2 are homologous to other BGWs that are upstream of *BEX *or *WEX *or *GASP *family genes, as assessed by whole genome BLASTn searches (*p *< 0.01); 1 is 5' to a neighbouring gene *HNRPH2 *(and is highly conserved in orthologous sequences in dog, rat and mouse); and, 2, including 1 5' of *GASP10ψ*, are not upstream of known coding transcripts, so might represent false positives, pseudogenic copies or longer-range regulators. We conclude that the BGW element is a conserved non-coding sequence motif shared by *BEX*, *WEX *and *GASP *genes, which is restricted to eutherian chromosome Xq21-22.

### Concerted evolution events within and between the 5' UTRs of *BEX*, *WEX *and *GASP *genes

**Figure 3:**
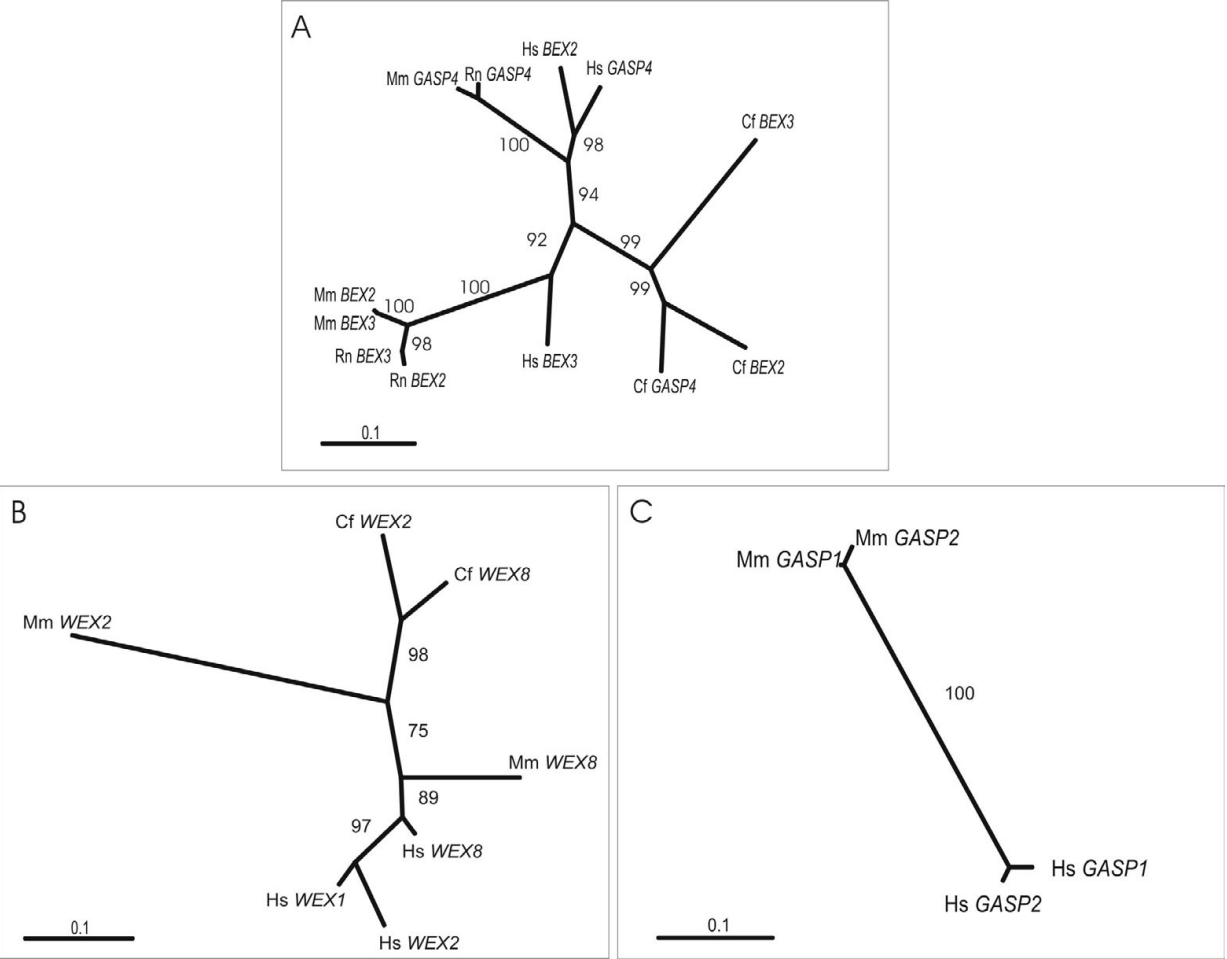
**Concerted evolution events among *BEX*, *WEX *and *GASP *genes. **Maximum likelihood phylogenetic trees for A) the first exon of the 5'UTR of *BEX2, BEX3 *and *GASP4 *genes B) the first exon of the 5' UTR of *WEX2*, *WEX8 *and *WEX1 *genes, and C) the exonic 5' UTR of *GASP1 *and *GASP2 *genes. 5' UTR sequences were extracted either from sequence transcripts or from homologous genomic regions. Maximum likelihood tree topologies and branch lengths were obtained using the program BASEML [54], for a given DNA sequence alignment. Each alignment was bootstrapped a 1000 times using the neighbour-joining method, and bootstrap values were overlaid the maximum likelihood tree. Abbreviations: Hs, *Homo sapiens*; Mm, *Mus musculus*; Rn, *Rattus norvegicus*; Cf, *Canis familiaris*.

Further investigation demonstrated that the evolutionary histories of portions of *BEX*, *WEX *and *GASP *genes were not identical to the relationships expected from the species' phylogenetic tree. The first exon of the 5' UTR of *BEX2*, *BEX3 *and *GASP4 *genes encompasses the BGW motif and exhibits greater sequence similarities between paralogues than it does between orthologues (Figure 3A). This finding is indicative of independent concerted evolution events [7,9,11,28,29]. Bootstrap values are sufficiently high to indicate with confidence that concerted evolution events have occurred among these genes in all mammalian lineages examined (human, mouse, rat and dog).

Notably, some of these concerted evolution events are lineage-specific. Bootstrap values (Figure 3A) support such events between human or dog *BEX2 *and *GASP4*, but not between mouse or rat *BEX2 *and *GASP4*, and between mouse, rat or dog *BEX2 *and *BEX3*, but not human *BEX2 *and *BEX3*.

Similarly, phylogenetic analysis of *WEX2 *and *WEX8 *genes indicates that concerted evolution events occurred recently, between the first 5' UTR exons, in the carnivore and primate lineages (Figure 3B). Finally, concerted evolution also occurred between the 5' UTRs of human or mouse *GASP1 *and *GASP2 *(Figure 3C).

Some of these concerted evolution events appear to have occurred relatively recently. In particular, concerted evolution between the 5' UTRs of mouse *Bex2 *and *Bex3 *(or rat *Bex2 *and *Bex3*) genes must have occurred very recently because they exhibit no substitutions of nucleotides within their BGW motifs (Figure 2).

### Chimaerism among *BEX*, *WEX *and *GASP *genes

Concerted evolution events between *BEX2*, *BEX3 *and *GASP4 *are restricted to the non-coding regions of their genes. The GASP4 protein exhibits no discernible sequence similarity to either BEX2 or BEX3 proteins; moreover, BEX2 and BEX3 amino acid sequences are relatively divergent (42% identity). Thus, these three genes appear to be chimaeric: their 5' UTRs are highly similar and exhibit a recent ancestry, whereas their protein coding sequences are more distantly related.

**Figure 4:**
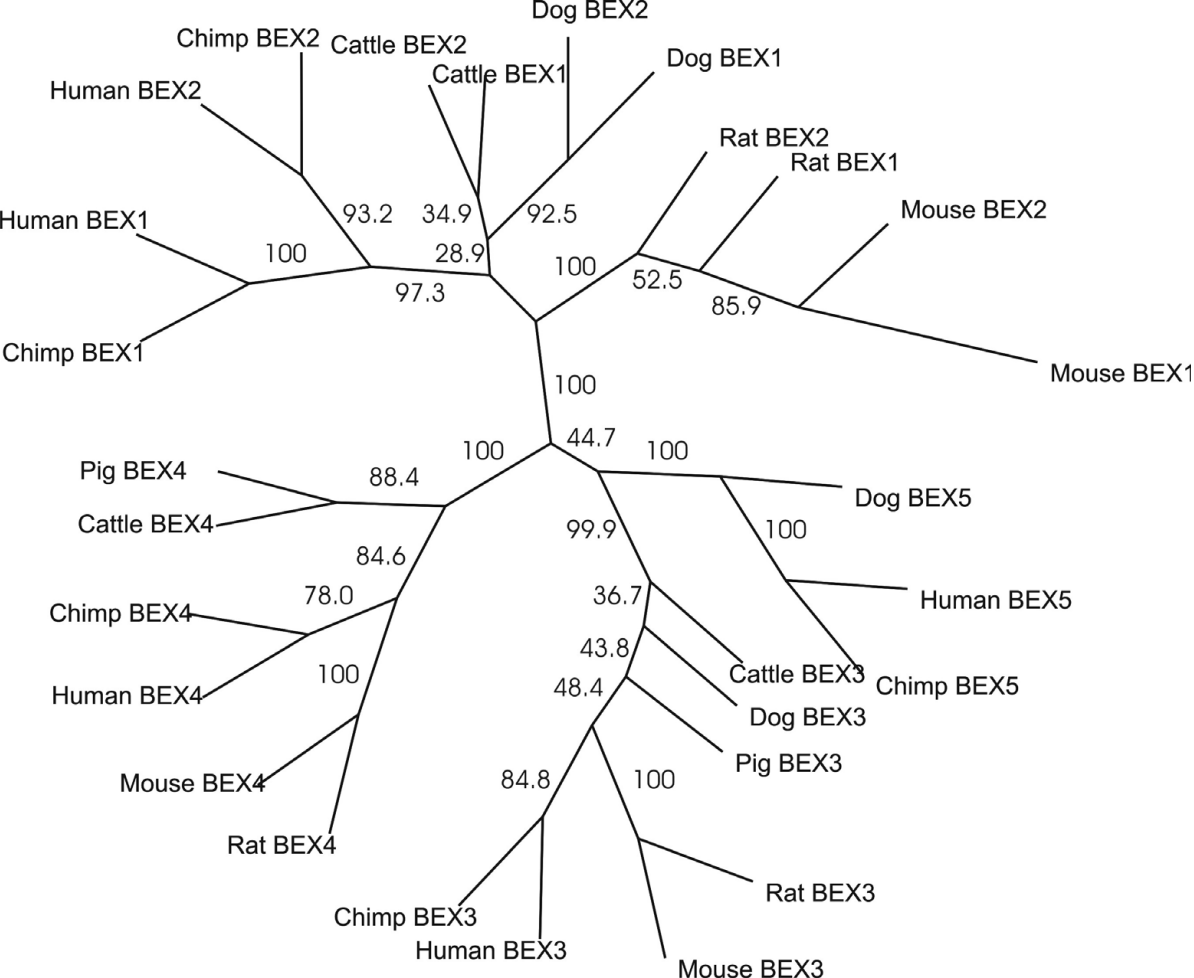
**Concerted evolution events among *BEX1 *and *BEX2 *protein-coding sequences. **A maximum likelihood phylogenetic tree for the *BEX *family members' protein-coding regions was constructed using the program BASEML [54] and a DNA sequence alignment. The alignment was bootstrapped a 1000 times using the neighbour-joining method, and provided bootstrap values, which were overlaid onto the maximum-likelihood tree.

This surprising conclusion is supported by a phylogenetic tree of BEX protein coding sequences (Figure 4). With high reliability the protein coding regions of human *BEX1 *and *BEX2 *paralogues were found to be more similar than they are to their rodent (mouse and rat), ruminant (cattle) or carnivore (dog) orthologues; similarly mouse *BEX1 *and *BEX2 *are most closely related, as are dog *BEX1 *and *BEX2 *genes. By contrast, the predicted evolutionary relationships of the three other paralogues, *BEX3*, *BEX4 *and *BEX5*, recapitulate the expected species tree [30].

These results thus demonstrate distinct and complex evolutionary histories for different regions of genes: concerted evolution events in the 5' UTRs are supported among *BEX2*, *BEX3 *and *GASP4*, while concerted evolution events in protein coding regions are supported only between BEX1 and BEX2. Among WEX proteins, a similar analysis indicates that WEX2, WEX4 and WEX8 coding sequences have experienced concerted evolution events (data not shown).

A pseudogene of human *GASP10 *(*GASP10ψ*), which is positioned upstream of GASP10 on chromosome X, appears to be converting with *GASP10 *in primate (human), rodent (mouse) and carnivore (dog) lineages. Bootstrap analysis supports that *GASP10 *and *GASP10ψ *are significantly more similar to each other than they are to their orthologues from dog or mouse (bootstrap value = 100%, data not shown). Therefore, although *GASP10ψ *does not encode a functional protein, it may function as a redundant copy for facilitating gene conversion events to its neighbouring GASP10 gene, as has occurred elsewhere in the human genome [31,32].

### Mechanism and mode of concerted evolution

These and similar inferences of concerted evolution (collated in Additional file 1) could have arisen due to unequal crossing-over, gene duplication or gene conversion. Unequal crossing-over and multiple gene duplications are unlikely mechanisms for concerted evolution of these genes because their orders and transcriptional orientations along their X chromosomes have suffered no rearrangements, inversions (except for one, involving rat *WEX2*) or large deletions when human, dog and mouse genomes are compared (Table 1; Figure 1; data not shown).

**Figure 5:**
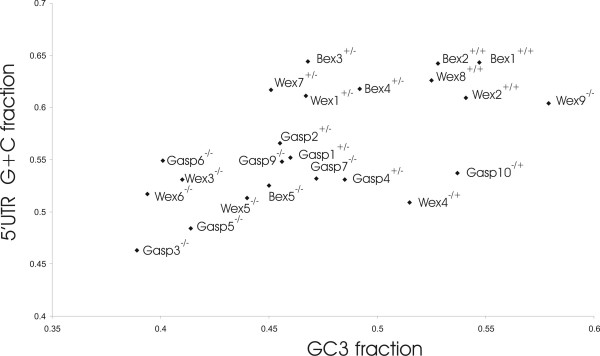
**G/C levels are elevated in human genes that undergo concerted evolution events. **5' UTR G+C and protein-coding GC3 properties of human *BEX*, *WEX *and *GASP *genes are indicated. (+) or (-) symbols represent presence or absence of a concerted evolution event in 5' UTR (first/left symbol) or in protein-coding (second/right symbol) regions.

The findings also could have arisen from exon shuffling, where exons are duplicated and inserted into different gene contexts [33]. However, inter-allelic gene conversion is distinguished from exon shuffling in that it often occurs within regions possessing high G+C nucleotide compositions [8-10,34]. Indeed, from G+C fractions for *BEX*, *WEX *and *GASP *5' UTRs and for the third positions of their protein coding regions ("GC3"), we found that sequences with evidence for concerted evolution possess significantly higher G+C fractions than sequences without such evidence (paired T-test, *p*(5' UTR) < 10^-3^, *p*(GC3) = 10^-3^) (Figure 5, Additional file 1). This indicates strongly that multiple events of interlocus gene conversion, rather than de novo sequence duplication or exon shuffling, have occurred among these genes.

### Evidence for sustained gene conversion events

Our findings indicate that the vertical transmission of *BEX*, *WEX *and *GASP *genes has been interrupted frequently by horizontal acquisitions of non-coding, as well as coding, sequences from genes that are closely-linked on human chromosome Xq22.1-q22.2. These genes' sequences appear to have been homogenized by multiple episodes of interlocus gene conversion. Because gene conversion is thought to proceed via formation of heteroduplexes between highly-similar sequences [35], this raises an interesting conundrum: how has recent gene conversion occurred between sequence-dissimilar genes drawn from different gene families?

**Figure 6:**
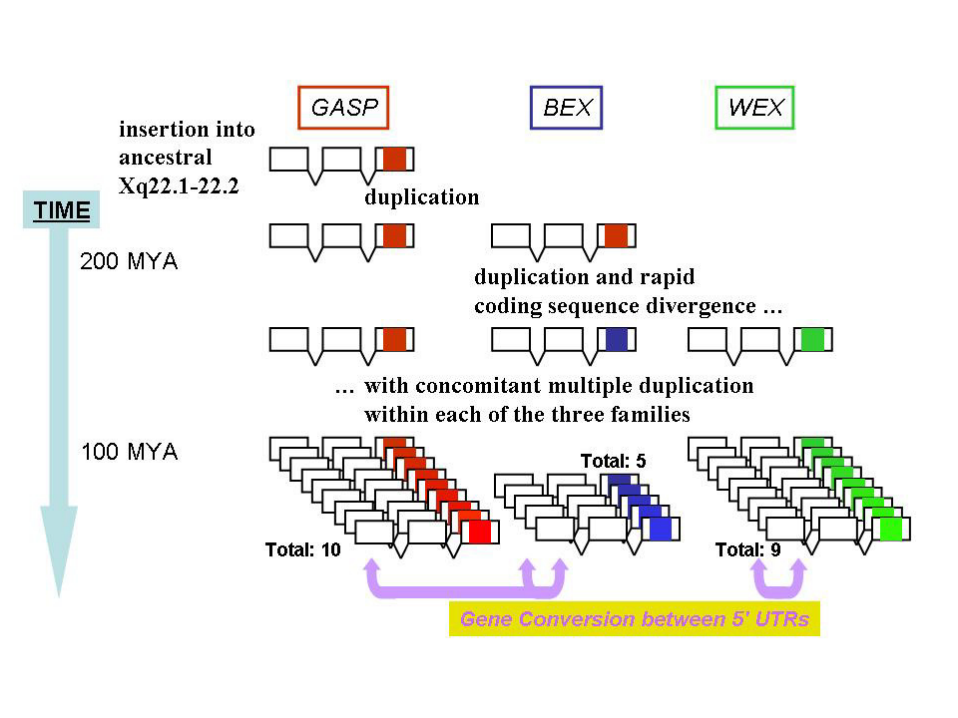
**Proposed evolutionary events leading to *BEX*, *WEX *and *GASP *genes on human chromosome Xq22.1-q22.2. ***BEX *and *WEX *genes arise due to duplication of a *GASP*-like gene early in eutherian mammalian history. These genes undergo multiple duplication events thereafter, but prior to the divergence of human and dog lineages. Multiple events of gene conversion, either between 5' UTRs or between coding sequences (Additional file 1), occur with the remaining coding sequence diverging rapidly due to relaxed selective constraints and/or adaptive evolution.

We resolve this question by proposing that BEX and WEX proteins all arose from a common ancestral *GASP*-like gene and rapidly diverged in sequence thereafter (Figure 6). We further propose that sequence similarity has been preserved between these homologues' 5' UTRs by recurrent episodes of gene conversion, despite rapid divergence of sequences elsewhere in their genes.

This scenario is supported by three observations: that *BEX *and *WEX *genes are homologous, that all three families utilize only a single exon to code for protein, and that *BEX*, *WEX *and *GASP *protein-coding sequences evolved rapidly. For the latter observation, we note that *K*_*A*_*/K*_*S *_values of these genes are high (median of 0.34) relative to a median value of 0.10 for all Ensembl human-mouse single orthologues (data not shown). These genes might then have arisen initially from duplication of *GASP10*, a gene that contains the BGW element and whose orthologues are known in earlier-diverging vertebrates, including fish (hypothetical protein FLJ20811; NM_212853). Unlike other GASP genes, *GASP10 *contains a three exon structure, similarly to these observed in *BEX *and *WEX *genes.

### Selection of gene conversion events

To our knowledge there is only a single documented occurrence of gene conversion between paralogues' 3' UTRs [36], and a single observation of gene conversion between paralogues' 5' UTRs [37]. However, multiple conversion events that differentiate between coding and non-coding sequences, as well as sequence conversion between genes that otherwise are not demonstrably homologous, are completely unexpected.

We attribute this singular evolutionary scenario to placental mammal-specific functional innovation. *BEX *and *WEX *genes appear, from the absence of homologues among other vertebrates, to have arisen during mammalian evolution by rapid sequence divergence from a common ancestor. In other analogous situations, such as the evolution of the caseins and histatins from a single ancestral gene [38], rapid gene duplication and sequence diversification is causally linked to innovation in physiology and behaviour [2]. In this case, gene function is associated with binding to brain-specific receptors, as seen for BEX and GASP proteins [15,20,21].

On the basis of frameshifts, stop codons, the lack of an initiating methionine codon or introns, all *BEX *and *WEX *paralogues outside of the Xq22.1-q22.2 region appear to be retrotransposed processed pseudogenes. This suggests that there is selection for retention of these genes as a closely-linked group on the X chromosome, and perhaps points either to gene conversion being a necessary requirement for long-term sustenance and evolution of their functions, or an X-linked factor that is necessary for their proper gene functions.

### Function prediction

The X chromosome contains a disproportionate number of genes related to mental functions which has been linked to the male preponderance of mental retardation cases [39,40]. However, of 9 X-linked genes that, when mutated, lead to mental impairment all possess orthologues in fish or even earlier-branching eukaryotes [40]. When expression information is available, all *BEX*, *WEX *and *GASP *genes are found to be expressed in the brain (Table 1). These eutherian-specific genes are thus possible candidates for the adaptive evolution of the neocortex, a region of the forebrain which is unique to mammals [41].

The presence of a conserved BGW element within the 5' UTRs of *BEX*, *WEX *and *GASP *genes is suggestive of its participation in regulation of translation. This is because translation rates have been shown previously to be affected by regulatory sequences, which include the start site consensus sequence, secondary structures, upstream AUGs, internal ribosome entry sites (IRES) and sequence specific recognition site for regulatory factors, such as protein or RNA [42,43]. Translational control of *BEX*, *WEX *and *GASP *genes might indicate that their proteins are utilized under specific physiological conditions [44], at developmental stages [45] or in subcellular compartments [46,47]. Another possible role for the BGW element might be to regulate alternative splicing. Although these genes possess only single protein coding exons there are several examples of transcripts that exhibit alternative splicing within their 5' UTR exons (e.g. *WEX2 *(mRNA BQ068054)) and others that exclude the protein-coding exon altogether (e.g. *GASP5 *(mRNA BC022066)).

## Conclusion

We have described the evolutionary history of a large region of human chromosome X, which appears to be an innovation of placental mammals. This region encompasses three previously unrelated protein-coding gene families, *BEX*, *WEX *and *GASP*, which have been the product of multiple gene duplications and large protein-coding sequence diversification since the earliest eutherian mammal. Despite the lack of protein-coding sequence similarity between many genes, we were able to identify a mammalian conserved DNA motif in their exonic 5'UTR, suggesting that they are derived of a common single ancestor, probably a *GASP*-like gene, found in early-branching bony vertebrates.

We have shown that the evolution of these paralogous genes has been affected by multiple events of gene conversion acting to homogenize among 5'UTR sequences, protein-coding sequences or both. Events of gene conversion in these regions have led to the occurrence of chimaeric genes, where their 5' UTRs are highly similar and exhibit a recent ancestry, but their protein coding sequences are more distantly related. We showed that the composition of sequences undergoing concerted evolution is enriched with G and C nucleotides, suggesting that biased gene conversion has been the underlying mechanism rather than exon shuffling.

*BEX*, *WEX *and *GASP *genes are found to be expressed in the brain (Table 1), suggesting that these eutherian-specific genes are possible candidates for the adaptive evolution of the neocortex, a region of the forebrain which is unique to mammal. The presence of a conserved BGW element within the 5' UTRs of *BEX*, *WEX *and *GASP *genes is suggestive of its participation in regulation of translation, possibly resulting in different spatio-temporal localization of these genes products or in different alternative splicing forms. These findings thus hint at hitherto unappreciated modes of 5' UTR evolution. The identification of such 5' UTRs elsewhere in the genome thus will be required as a contribution to the delineation of all human sequence under selection.

## Methods

### Genome assemblies and gene models

The July 2003 human (based on NCBI Build 34), the October 2003 mm4 *Mus musculus *genome assembly (based on NCBI Build 32), the rn3 June 2003 *Rattus norvegicus *genome assembly (based on version 3.1), the canFam1 July 2004 *Canis familiaris *whole genome shotgun (WGS) assembly v1.0, the 13 Nov. 2003 chimpanzee (*Pan troglodytes*) Arachne assembly – NCBI Build 1 Version 1, and the February 2004 chicken (*Gallus gallus*) draft assembly were analysed. Gene annotations were extracted from the UCSC genome browser [48], or were predicted using Genewise [49] and available transcriptional information.

### BGW element

The ENSEMBL web browser blast application [50] and the BlastN program [24] (without optimization for identical hits) were used to perform a sequence similarity search between a region 5' to Alex2 (chrX:99686693–99686992) and the entire human genome assembly. Multiply aligned genomic hits that were predicted (*p *< 0.01) to be homologous to this region were positioned on human X chromosome, only within an area containing *BEX*, *WEX*, and *GASP *family members. A similar search was done in mouse, rat, zebrafish and chicken genomes. Human, mouse and rat sequences were thereafter multiply aligned, and used to further search the human genomic area (chrX:99850000–100250000) using an HMM [51]. Additional similar sequences (*E *< 0.1) were added to generate the final multiple alignment (Figure 2).

### Tree construction and bootstrap analysis

Protein-coding and exonic 5' UTR phylogenetic trees were both constructed from DNA sequence alignments. Sequences were aligned using ClustalW [52] or MULAN [53], and alignments were subjected to a neighbour-joining bootstrapping process (*n *= 1000). Non protein-coding branch length estimations were calculated using a maximum likelihood approach (BASEML [54]) as implemented by the molecular evolution package DAMBE [55]. In order to assign bootstrap values for the branch lengths, neighbour-joining bootstrap values were superimposed on the ML tree.

### G+C and GC3 content analysis

G+C proportions contained within the exonic-5' UTR or the entire 5' UTR were based on genomic sequences. GC3 content was calculated from examining the GC fraction of the third nucleotide codon positions of protein-coding sequences.

### Exonic 5' UTR sequence comparisons

The longest 5'-UTR sequence of each gene was chosen using all available mRNA and EST transcript information (Table 1). In all cases, sequences were further extended by 300 bases, using genomic data, to account for foreshortened transcript evidence. UTR sequences were then aligned using BLASTN and considered to be homologous when *p *< 0.001.

### *K_A_*/*K_S _*analysis

Ratios of *K*_*A *_(the number of nonsynonymous substitutions per nonsynonymous site) to *K*_*S *_(the number of synonymous substitutions per synonymous site) were calculated using the yn00 method of Yang and Nielsen [56].

## Authors' contributions

EEW identified the shared homology between the 5'UTR of BEX and GASP genes and generated much of the analysis. CPP identified the BGW motif, and assisted in data interpretation. EEW and CPP both contributed to the drafting of the manuscript.

## Supplementary Material

Additional File 1 **Evidence for gene conversion events among mammalian *BEX*, *WEX *and *GASP *genes. **Sequences were grouped according to the gene region they encompass: exonic 5' UTR, 5' UTR (exons and introns) and protein-coding regions. Validation method: (a) A phylogenetic tree indicating significantly greater proximity between gene paralogs in a particular genome than for gene orthologous of different organisms' genomes. Neighbour-joining bootstrap values for the tree topology were considered if they were greater than 85% (b) Sequence identity levels between paralogs are substantially higher than sequence identity levels between orthologs (>85% compared with <65%, respectively). Abbreviations: N/D, not determined. Click here for file
