# Supplementary material for: Mammalian BEX, WEX and GASP genes: Coding and non-coding chimaerism sustained by gene conversion events
Source: BMC Evol Biol. 2005 Oct 12;5:54. doi: 10.1186/1471-2148-5-54 (PMC1274310; doi:10.1186/1471-2148-5-54)
Supplement: Additional File 1 — Evidence for gene conversion events among mammalian BEX, WEX and GASP genes. Sequences were grouped according to the gene region they encompass: exonic 5' UTR, 5' UTR (exons and introns) and protein-coding regions. Validation method: (a) A phylogenetic tree indicating significantly greater proximity between gene paralogs in a particular genome than for gene orthologous of different organisms' genomes. Neighbour-joining bootstrap values for the tree topology were considered if they were greater than 85% (b) Sequence identity levels between paralogs are substantially higher than sequence identity levels between orthologs (>85% compared with <65%, respectively). Abbreviations: N/D, not determined. [file 1471-2148-5-54-S1.doc]

| **Gene name** | **5’ UTR (exons)** | **5’UTR** | **Protein-coding** |
| --- | --- | --- | --- |
| Gasp4 | + (a) | - | - |
| Gasp7 | - | - | - |
| Gasp10ψ | - | - | + (a) |
| Gasp10 | - | - | + (a) |
| Gasp6 | - | - | - |
| Gasp9 | - | - | - |
| Gasp5 | - | - | - |
| Gasp1 | + (a) | + (b) | - |
| Gasp2 | + (a) | + (b) | - |
| Gasp3 | - | - | - |
| Bex1 | + (b) | + (b) | + (a) |
| Bex2 | + (a) | + (b) | + (a) |
| Bex3 | + (a) | + (b) | - |
| Bex4 | + (b) | + (b) | - |
| Wex1 | N/D | N/D | - |
| Wex2 | + (a) | + (b) | + (a) |
| Wex3 | - | - | - |
| Wex4 | - | - | + (a) |
| Wex5 | - | - | - |
| Wex6 | - | - | - |
| Wex7 | N/D | N/D | - |
| Wex8 | + (a) | + (b) | + (a) |
| Wex9 | - | - | - |
